# Photocatalytic hollow TiO_2_ and ZnO nanospheres prepared by atomic layer deposition

**DOI:** 10.1038/s41598-017-04090-0

**Published:** 2017-06-28

**Authors:** Nóra Justh, László Péter Bakos, Klára Hernádi, Gabriella Kiss, Balázs Réti, Zoltán Erdélyi, Bence Parditka, Imre Miklós Szilágyi

**Affiliations:** 1 Department of Inorganic and Analytical Chemistry, Budapest University of Technology and Economics, H-1111 Budapest, Hungary; 2 Department of Applied and Environmental Chemistry, University of Szeged, H-6720 Szeged, Hungary; 3 Department of Solid State Physics, University of Debrecen, H-4026 Debrecen, Hungary; 4 MTA-BME Technical Analytical Chemistry Research Group, H-1111 Budapest, Hungary

## Abstract

Carbon nanospheres (CNSs) were prepared by hydrothermal synthesis, and coated with TiO_2_ and ZnO nanofilms by atomic layer deposition. Subsequently, through burning out the carbon core templates hollow metal oxide nanospheres were obtained. The substrates, the carbon-metal oxide composites and the hollow nanospheres were characterized with TG/DTA-MS, FTIR, Raman, XRD, SEM-EDX, TEM-SAED and their photocatalytic activity was also investigated. The results indicate that CNSs are not beneficial for photocatalysis, but the crystalline hollow metal oxide nanospheres have considerable photocatalytic activity.

## Introduction

Photocatalysis using solar energy has been acknowledged as an environment friendly method to degrade pollutants and to treat wastewater^1^. Titanium dioxide and zinc oxide are widely used as photocatalysts in many reactions due to their chemical stability, non-toxicity and high reactivity^2–6^. However, the fast electron–hole recombination of the photo-excited charge carriers leads to their short lifetime in the oxides. In addition ZnO and TiO_2_ they have narrow light response range limited to UV due to the large bandgap of TiO_2_ (3.2 eV) and ZnO (3.3 eV). These limitations interfere with achieving maximum activity of the photocatalysts; thus, it is desirable to use a co-catalyst to synthesize photocatalysts with improved charge separation, low recombination rates and wider response ranges^4, 7–9^. Carbon-based nanomaterials (e.g. nanotubes, nanospheres, fullerenes, graphene) are very attractive due to their high surface area, good thermal and electrical conductivity, mechanical as well as chemical stability, and they can be ideal co-catalysts in carbon-metal oxide composites^10, 11^. Among them, carbon nanospheres (CNSs) have the unique feature that they can be used as templates to produce inorganic hollow spheres, such as ZnO and TiO_2_, which have special optical, optoelectronic, magnetic, electrical, thermal, electrochemical, photoelectrochemical and catalytic properties^12–18^. There are many ways to synthesize carbon nanospheres, for example laser ablation, chemical vapor deposition and hydrothermal methods, the latter of which is a simple and easy tool to prepare CNSs in large quantitites^11, 19–23^. Metal oxides then can be deposited onto the carbon carriers with numerous techniques^24^, from which atomic layer deposition (ALD) is an outstanding method to prepare carbon-metal oxide composites, since it allows the coating of the surface of nanostructures in a conformal and homogeneous way, with nanoscale precise control of the thickness of the deposited film. ALD of TiO_2_ and ZnO was already performed successfully on graphene and carbon nanotubes, but deposition on CNSs has not yet been reported to the best of our knowledge^10, 25–28^.

The goal of our research was to deposit semiconductor metal oxide nanolayers on the surface of carbon nanospheres, and to subsequently burn out the carbon cores to get hollow metal oxide nanospheres. The carbon nanospheres, the carbon-metal oxide composites and the hollow metal oxide nanospheres were characterized by thermogravimetry/differential thermal analysis coupled with mass spectrometry (TG/DTA-MS), Fourier-transformation infrared spectroscopy (FTIR), Raman spectroscopy, powder X-ray diffraction (XRD), scanning electron microscope - energy-dispersive X-ray spectroscopy (SEM-EDX), transmission electron microscope - selected area electron diffraction (TEM-SAED), and the photocatalytic activity of the samples was also investigated.

## Results and Discussion

### Thermal analysis

**Figure 1:**
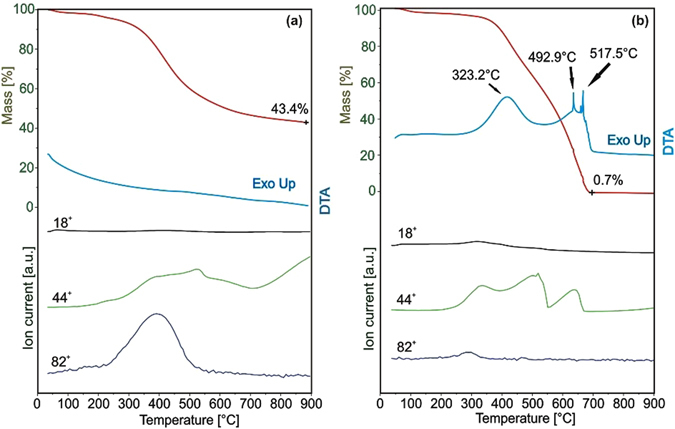
TG/DTA-MS measurements of the carbon nanospheres. (**a**) in helium, (**b**) in air atmosphere. The molecule ions were associated with water (18^+^), carbon dioxide (44^+^) and cyclohexene (82^+^).

Figure 1a shows the TG/DTA-MS analysis of the pure carbon nanospheres in helium atmosphere. The decomposition begins around 300 °C, which can be seen from the evolving gases of CO_2_ (m/z = 44) and cyclohexene (m/z = 82), and 43.4% of the mass remained at 900 °C. This ensured that the ALD depositions could be done safely at 250 °C without damaging the template. Figure 1b displays the thermal analysis of the carbon spheres in air atmosphere, the functional groups left at 323.2 °C, and the remaining carbon structure degraded at 492.9 °C and 517.5 °C, and burned out completely at 700 °C, leaving behind only 0.7% of the mass. These processes were accompanied by exothermic heat effect due to the combustion of the organic material. The results mean that heating the core-shell composites to 700 °C would remove the template, leaving only the hollow metal oxide spheres behind (Fig. S1). The molecule ion of CO_2_ (44^+^) was dominant in the mass spectrum of the evolved gaseous products because of oxidation, and in contrast to the analysis in helium, the cyclohexene was detected to a smaller amount^8, 13, 29, 30^.

### Formation of the core-shell and hollow nanospheres

The parameters of the ALD reactions are shown in Table 1 for each sample. On the carbon nanospheres two photocatalytic semiconductor oxides, i.e. TiO_2_ and ZnO were grown. In the case of the TiO_2_ we applied two different temperatures, 80 °C to grow amorphous TiO_2_, and 250 °C to obtain crystalline TiO_2_; these temperatures were selected from previous measurements of our group. The ZnO growth rate is higher than the growth rate of TiO_2_ hence fewer cycles were used in the case of the deposition of ZnO^31^. The approximate shell thickness and bandgap data was obtained by UV-VIS reflection. The hollow metal oxide nanospheres were created by heating the composites to 700 °C. In the name of the specimens, the C means the composite core-shell material and the H stands for the hollow spheres obtained after burning out the carbon core.

**Table 1:** Parameters of the ALD process, shell thicknesses and bandgaps.

Sample name	Type	Deposited oxide	Temperature	Number of cycles	Pulse times	Shell thickness	Bandgap
C-TiO_2_-80C	Composite	TiO_2_	80 °C	700	0.3 s TiCl_4_-3 s N_2_/0.3 s H_2_O-3 s N_2_	9.0 nm	3.06 eV
H-TiO_2_-80C	Hollow						
C-TiO_2_-250C	Composite	TiO_2_	250 °C	700	0.3 s TiCl_4_-4 s N_2_/0.45 s H_2_O-3 s N_2_	19.7 nm	3.07 eV
H-TiO_2_-250C	Hollow						
C-ZnO-250C	Composite	ZnO	250 °C	100	0.3 s Et_2_Zn-3 s N_2_/0.3 s H_2_O-3 s N_2_	49.3 nm	3.07 eV
H-ZnO-250C	Hollow						

### FTIR and Raman spectroscopy

**Figure 2:**
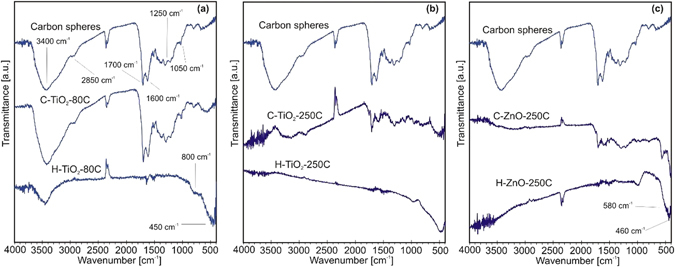
FTIR spectra of the samples. The spectra of the bare CNSs, the composite and the hollow nanospheres are shown with (**a**) TiO_2_ deposited at 80 °C, (**b**) TiO_2_ deposited at 250 °C and (**c**) ZnO deposited at 250 °C.

In the FTIR spectra (Fig. 2) at 1600 cm^−1^ the stretching of the carbon-carbon double bonds can be seen, and at 2850 cm^−1^ the bending of the carbon-hydrogen bonds are visible^11^. The main stretching bands of the carbonyl group (C = O) are at 1700 cm^−1^, while the epoxy group (C-O-C) were detected at 1250 cm^−1^ and the C-O vibrations at 1050 cm^−1^ ^32^. At 3400 cm^−1^ the characteristic vibrations (bending and stretching) of the OH groups are present^33^. The lattice vibration bands of the TiO_2_ (Fig. 2a and b, at 800 and 450 cm^−1^) and the ZnO (Fig. 2c, at 580 and 460 cm^−1^) are under 1000 cm^−1^, which are best visible on the spectra of the hollow shells, since due to their low amount, they are less observable on the spectra of the composites^34, 35^.

**Figure 3:**
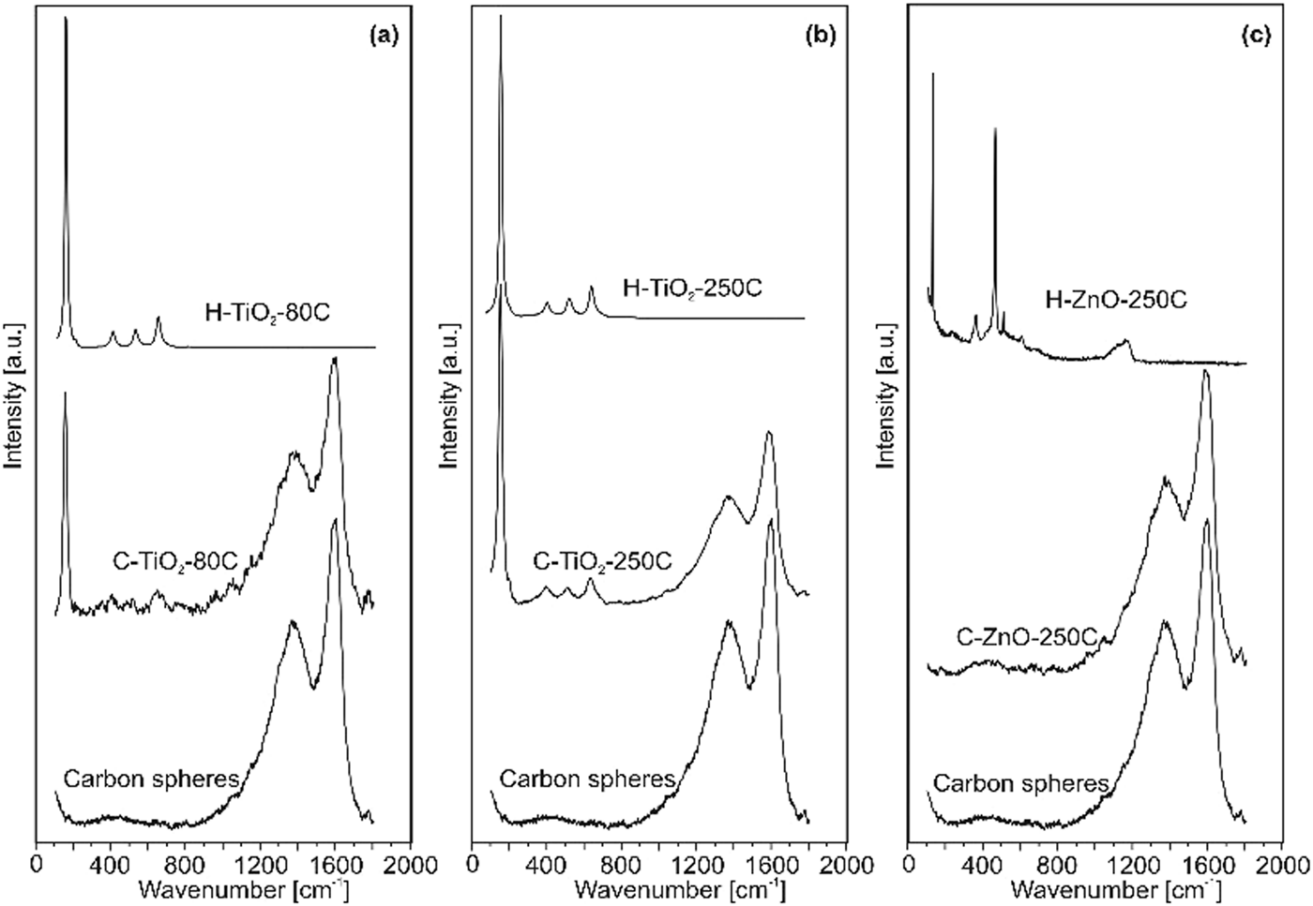
Raman spectra of the samples. The spectra of the bare CNSs, the composite and the hollow nanospheres are shown with (**a**) TiO_2_ deposited at 80 °C, (**b**) TiO_2_ deposited at 250 °C and (**c**) ZnO deposited at 250 °C.

In the Raman spectra of the specimens (Fig. 3), the characteristic vibrations of the carbon can be seen at 1580-1600 cm^−1^ (G band) and 1350 cm^−1^ (D band), which are not present in the case of the hollow spheres, indicating that the removal of the carbon template was successful (as shown in the FTIR spectra as well (Fig. 2))^11, 19^. The most intense peak of the anatase TiO_2_ (Fig. 3a and b) is at 141 cm^−1^, and its other three peaks are at around 400, 516 and 637 cm^−1^ on the spectra of the composite and hollow samples^36^. In Fig. 3c, the peaks of the ZnO at 101, 377, 409, 436, and 1200 cm^−1^ are visible again the best in the case of the hollow ZnO wurtzite spheres^37^.

### Powder XRD and SAED measurements

**Figure 4:**
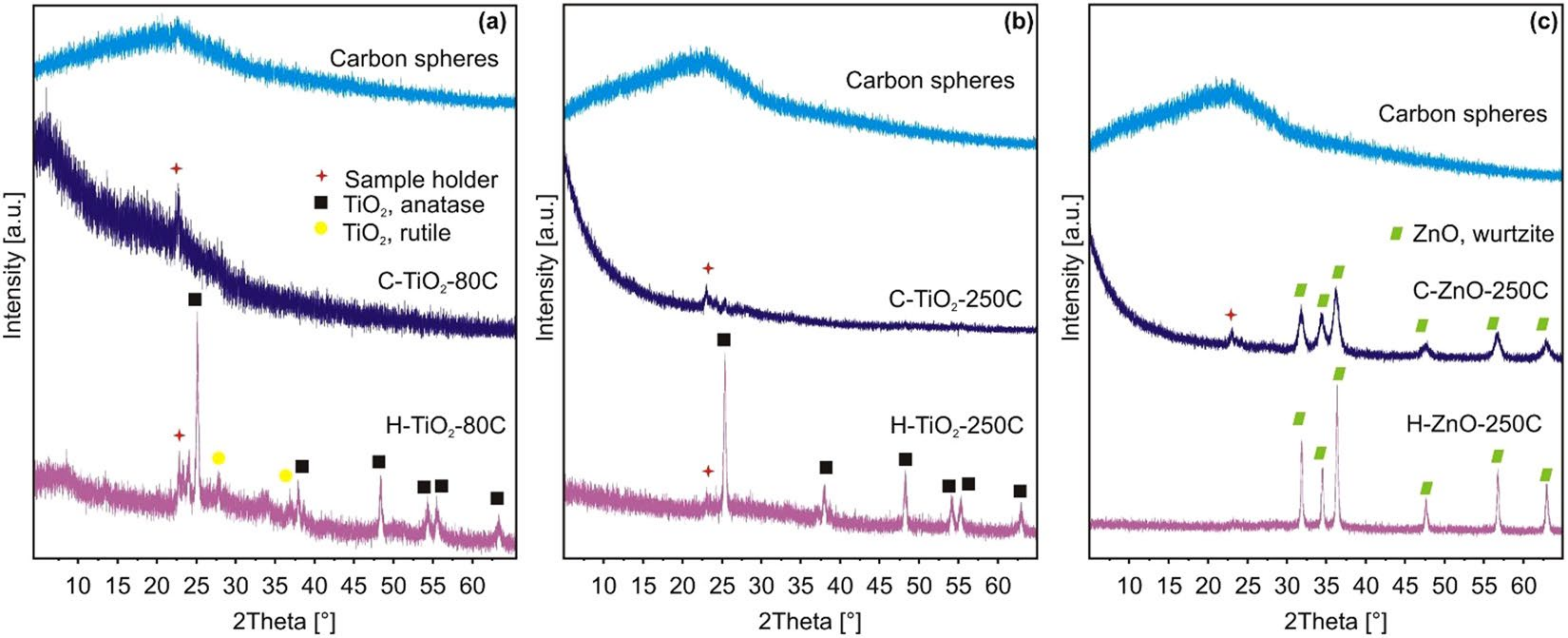
XRD diffractograms of the specimens. The diffractograms of the bare CNSs, the composites and the hollow nanospheres are shown with (**a**) TiO_2_ deposited at 80 °C, (**b**) TiO_2_ deposited at 250 °C and (**c**) ZnO deposited at 250 °C.

**Figure 5:**
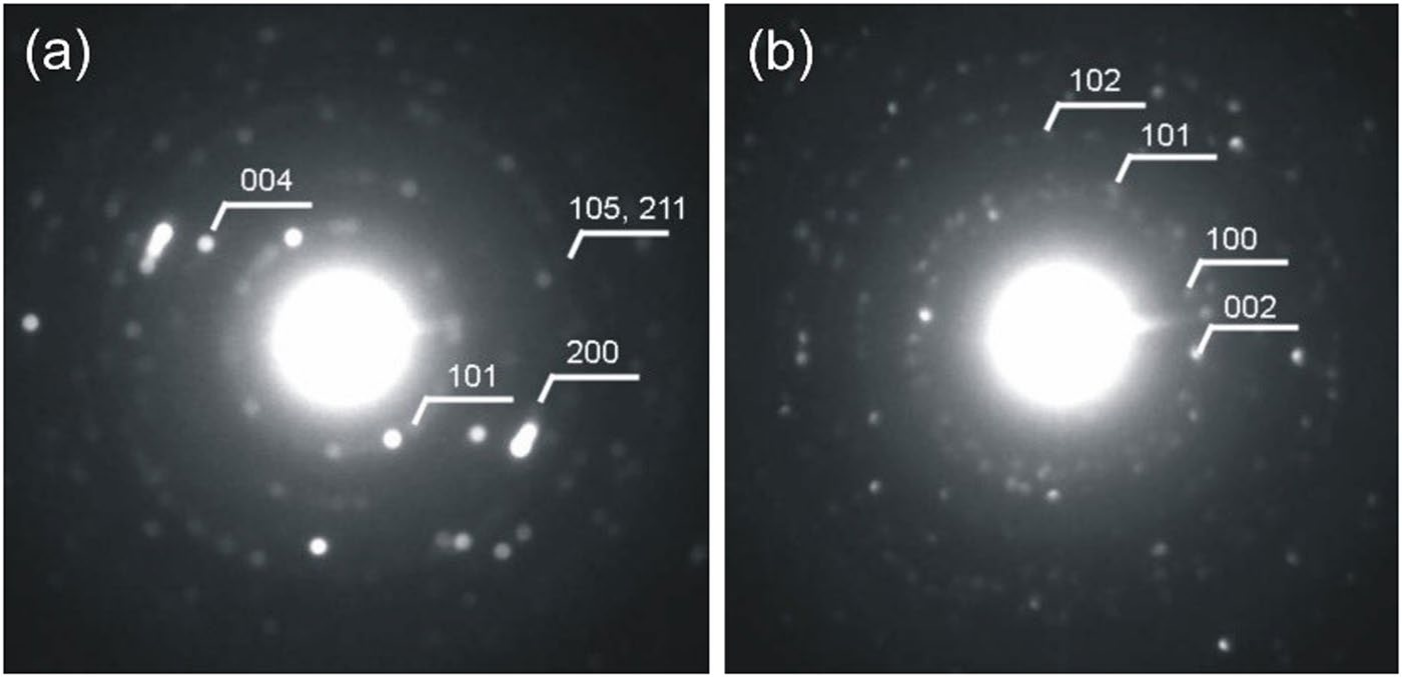
Electron diffraction of the hollow spheres. (**a**) H-TiO_2_-250C and (**b**) H-ZnO-250C. The numbers are corresponding to the Miller indices of the TiO_2_ and ZnO, respectively.

Figure 4 shows the powder X-ray diffractograms of the samples. The pure carbon spheres and the carbon-TiO_2_ composites (Fig. 4a and b) are amorphous, while the peaks of the crystalline ZnO can be seen on the carbon-ZnO composites (Fig. 4c). All hollow oxide spheres are crystalline: the H-TiO_2_-80C contained 84% anatase (ICDD 01-075-2546) and 16% rutile (ICDD 01-088-1173), the H-TiO_2_-250C was identified as pure anatase and the H-ZnO-250 was hexagonal zinc-oxide (ICDD 01-080-4199). The small peaks around 2Theta = 23° come from the sample holder. The electron diffraction patterns of samples H-TiO_2_-250C and H-ZnO-250C also confirm that the hollow spheres are crystalline (Fig. 5).

### SEM-EDX

The composition of the carbon nanospheres, carbon-metal oxide composites and hollow oxide nanospheres calculated from EDX spectra are shown in Table 2 (Fig. S2). The results follow the synthesis process: the carbon spheres contain only carbon and oxygen; after the ALD the metal content appears and the subsequent burning out of the template removes the carbon. The chlorine is residue from the TiCl_4_ ALD precursor.

**Table 2:** Composition of the samples from EDX measurements.

Sample	C	O	Ti	Cl	Zn
	atomic %				
Carbon spheres	77.3	22.7			
C-TiO_2_-80C	76.1	23.2	0.7	0.0	
H-TiO_2_-80C	0.0	72.0	27.6	0.4	
C-TiO_2_-250C	57.4	34.5	8.0	0.1	
H-TiO_2_-250C	0.0	76.3	23.6	0.1	
C-ZnO-250C	68.6	25.9			5.5
H-ZnO-250C	0.0	39.9			60.1

**Figure 6:**
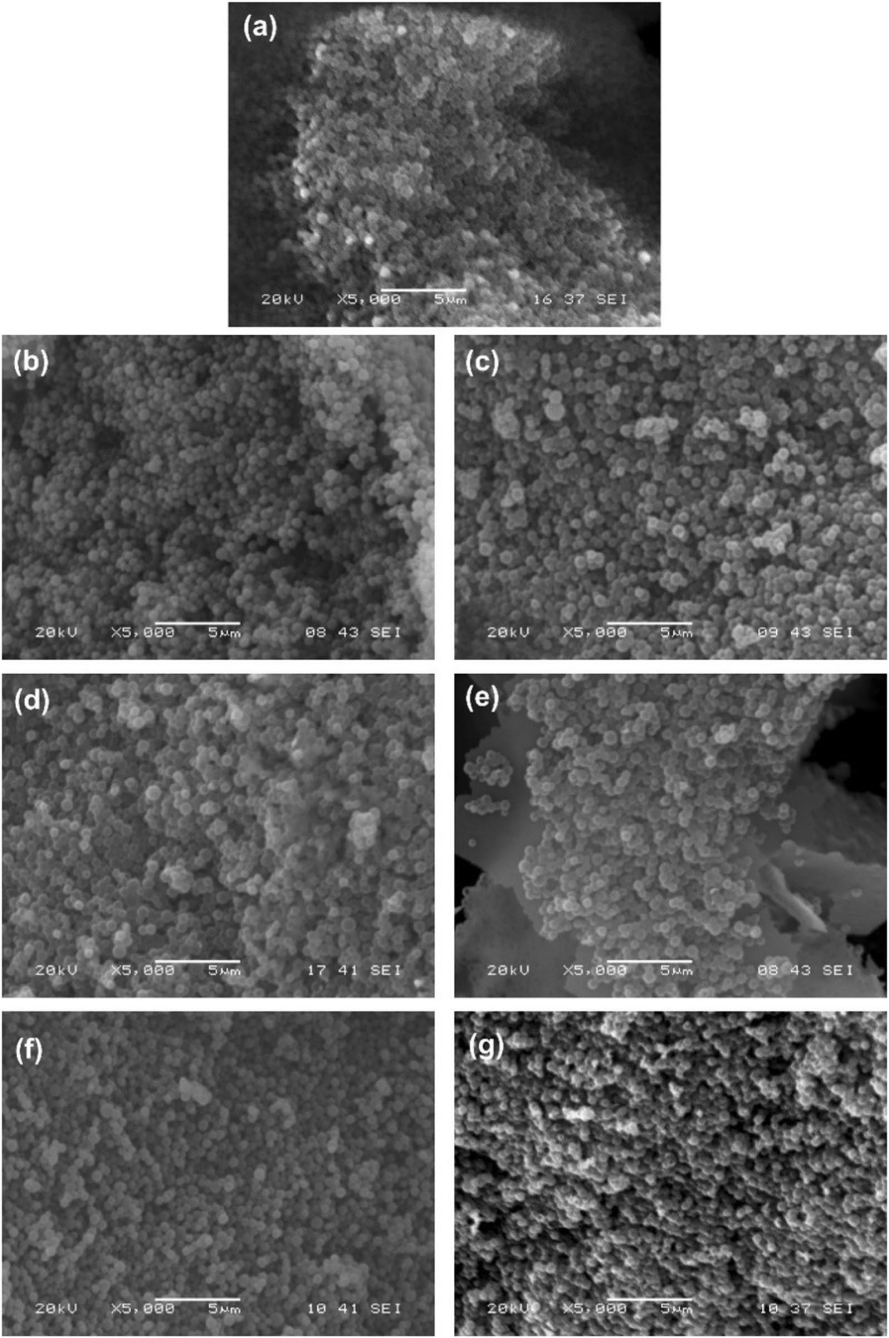
SEM images of the samples. (**a**) pure carbon spheres, (**b**) C-TiO_2_-80C, (**c**) H-TiO_2_-80C, (**d**) C-TiO_2_-250C, (**e**) H-TiO_2_-250C, (**f**) C-ZnO-250C and (**g**) H-ZnO-250C.

In Fig. 6a, the SEM images of the carbon nanospheres show their spherical shape and relatively uniform size distribution. From measurement of 100 bare carbon spheres their mean diameter is 547 nm with a deviation of 88 nm. The spherical shape was retained both after the ALD and the burning out of the carbon templates too (Fig. 6b-g).

### TEM

**Figure 7:**
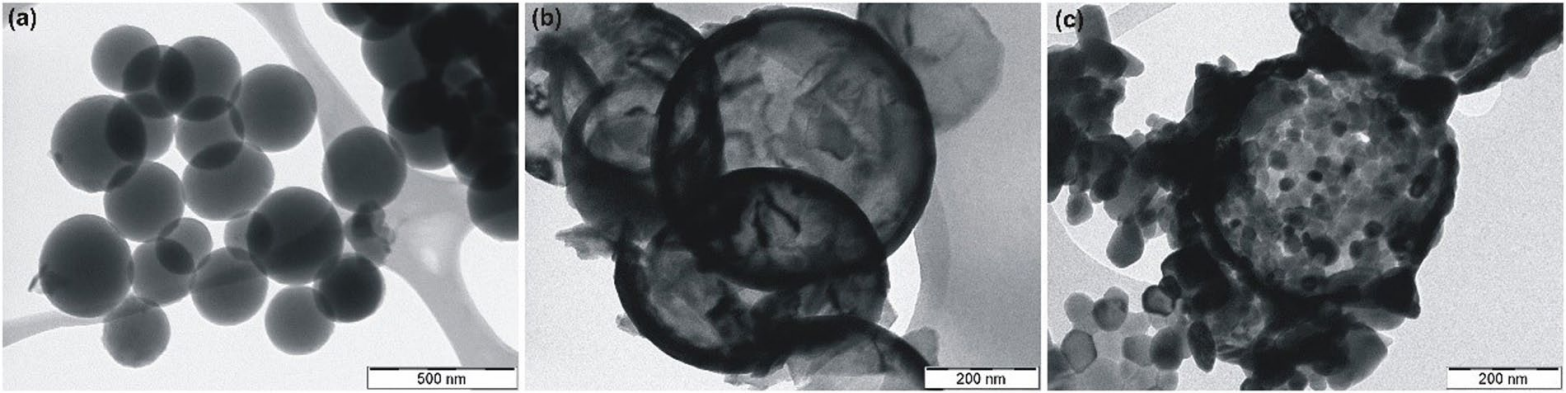
TEM images of the specimens. (**a**) C-TiO_2_-80C, (**b**) H-TiO_2_-250C and (**c**) H-ZnO-250C.

Figure 7a demonstrates the TEM image of composite spheres, and Fig. 7b and c shows the hollow samples, where the hollow nature of the specimens is visible. The shell thicknesses from these images are comparable to the UV-VIS measurements (Table 1). Some shells are broken due to the sample preparation for TEM, which was ultrasound sonication in ethanol.

### Photocatalysis

**Figure 8:**
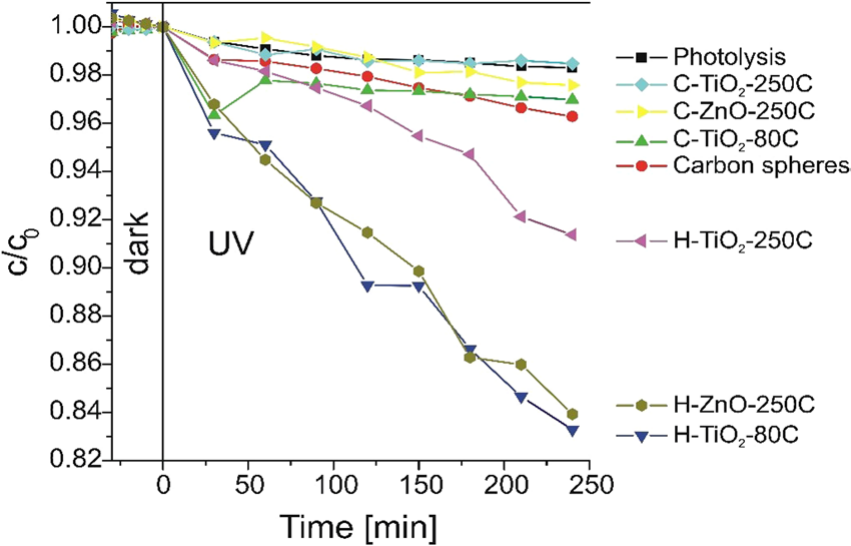
Photocatalytic activity of the samples.

The results of the photocatalytic activity are shown in Fig. 8. The pure carbon nanospheres and carbon-metal oxide composites have no significant photocatalytic effect, the relative absorbance only decreases slightly compared to the photolysis of the bare methyl orange sample after four hours of UV light radiation. This shows that the CNSs are not beneficial as co-catalyst for ALD metal oxide photocatalyst. According to previous results, carbon nanospheres can be beneficial for enhancing TiO_2_ photocatalysis^8^. We assume that in our case the CNSs behave as insulators rather than semiconductors due to the large number of heteroatoms, which impair the photocatalytic effect of the TiO_2_. The hollow metal oxide spheres have clearly beneficial effect on the photocatalytic degradation of the methyl orange dye. The photocatalytic property of the H-TiO_2_-80C sample is much better than the H-TiO_2_-250C, which can be seen from the apparent rate constant (k_app_) of the photocatalytic activity (Table 3), which was the slope of the −ln(c/c_0_) - time relation by assuming pseudo first order reaction kinetics. One reason for this is that the H-TiO_2_-80C spheres contained rutile beside the anatase phase, which enhances photocatalysis due to their difference in the band position, in the indirect-direct nature of their bandgaps, and the effect of the solid-solid interface^38^. The other reason is that the H-TiO_2_-80C samples have thinner shells (Table 1) because of the ALD parameters (lower temperature and shorter N_2_ purge and H_2_O pulse times), while their size is similar, hence the specific surface area is greater in the hollow shells. This is further proven by the fact that after burning out the carbon template from these samples (Figure S1), according to the TG/DTA data, only 7.95% mass remains in the case of H-TiO_2_-80C, which equals to its TiO_2_ content. However, at the specimen H-TiO_2_-250C this mass is 30.37%. From the sample H-ZnO-250C, 42.04% remains after annealing; therefore its specific surface area is even smaller, albeit its photocatalytic effect is almost the same as the effect of the H-TiO_2_-80C, because in this case ALD ZnO can be a better photocatalyst than ALD TiO_2_^39^. TiO_2_ and ZnO was also deposited on flat silicon wafers with the same parameters as on the CNSs and their photocatalytic activity was investigated (Fig. S3). They showed considerably lower photocatalytic activity, the reason for this is the smaller specific surface area of the flat surface compared to the nanospheres.

**Table 3:** k_app_ of the hollow shell photocatalysts.

Sample	k_app_ [10^−4^ min^−1^]
H-TiO_2_-80C	7.979
H-TiO_2_-250C	3.456
H-ZnO-250C	7.530

## Conclusion

We managed to prepare carbon nanospheres with a hydrothermal method. They were successfully coated for the first time with TiO_2_ and ZnO layers by atomic layer deposition. The TiO_2_ was amorphous and the ZnO was crystalline after the ALD. The subsequent removal of the CNSs created the hollow metal oxide nanospheres, which were all crystalline. CNSs proved to be not beneficial for photocatalysis due to the presence of heteroatoms, as they did not degrade significantly the methyl orange dye. The hollow nanospheres possess considerable photocatalytic activity, which is better with thinner shells in the case of TiO_2_.

## Methods

### Synthesis of the carbon nanospheres

The carbon nanospheres were prepared by a hydrothermal method as follows^11^. 0.15 M sucrose solution was placed into an autoclave with a volume of 175 cm^3^. The pH was set to 11 using 0.194 M NaOH solution. The reaction went for 12 hours at 180 °C under autogenous pressure. The resulting product was washed with warm distilled water until the dark yellow color of the filtrate faded. After that, the sample was washed with 5, 15, 45 V/V % ethanol-water mixture, three times with each. This was followed by three more washes with warm distilled water. Finally, it was placed in a drying cabinet at 70 °C for overnight. The resulting product was a fine black powder^40^.

### Atomic layer deposition of the metal oxides on the carbon nanospheres

A Beneq TFS-200-186 ALD thermal reactor was used at 1 mbar pressure to conduct the atomic layer deposition. Layers of TiO_2_ and ZnO were made with the reaction of TiCl_4_ and (C_2_H_5_)_2_Zn with H_2_O, respectively. The parameters of the deposition are shown in Table 1.

### Preparation of the hollow metal oxide nanospheres

The carbon nanospheres-metal oxide composites were heated to 700 °C in a TA Instruments SDT 2960 simultaneous TG/DTA-MS device in air atmosphere (130 ml/min) using an open platinum crucible and 10 °C/min heating rate.

### Characterization

TG/DTA-MS measurements were conducted in the above mentioned device in helium and air atmospheres (130 ml/min). Evolved gas analytical (EGA) MS curves were recorded by a Balzers Instruments Thermostar GSD 200 T quadruple mass spectrometer coupled on-line to the TG/DTA instrument. The on-line coupling between the two parts was provided through a heated (T = 200 °C), 100% methyl deactivated fused silica capillary tube with an inner diameter of 0.15 mm.

UV-VIS reflection for film thickness and bandgap determination was measured with an Avantes AvaSpec-2048 Fiber Optic spectrometer. Table S1 contains the thicknesses of the reference oxide films deposited on flat silicon wafers.

FTIR measurements were carried out between 4000 and 400 cm^−1^ on a Biorad Excalibur Series FTS 3000 infrared spectrometer, in KBr pellets.

Raman spectra were made by using a Jobin Yvon Labram Raman instrument equipped with an Olympus BX41 microscope. The laser was frequency duplicated green Nd-YAG with 532 nm wavelength. The spectra were taken from 100 to 1800 cm^−1^.

Powder XRD patterns were recorded on a PANanalytical X’Pert Pro MPD X-ray diffractometer using Cu Kα radiation. Crystalline phases were identified and their ratio was calculated by X’Pert HighScore Plus software.

SEM-EDX data were obtained by a JEOL JSM-5500LV scanning electron microscope. The specimens were fixed on the Cu/Zn alloy sample holders with carbon tape, and were sputtered with an Au/Pd conductive layer for the imaging. The average composition in atomic % from EDX spectra was calculated from three measurements on each sample.

TEM-SAED images were made on a FEI Morgagni 268 device.

Photocatalytic activity of the samples were investigated by putting 1.0 mg of them together with 3 ml aqueous solution of methyl orange dye (4 × 10^−5^ M) in quartz cuvettes. The flat samples were deposited on 0.8 × 1.8 cm^3^ silicon wafers, and the wafers was immersed in the solution. After waiting one hour for the adsorption equilibrium, the cuvettes were placed between two parallel Osram 18 W blacklight lamps (spectrum is in Figure S4), 5 cm from each, and were measured every half hour with a Jasco V-550 UV-VIS spectroscope for four hours. The decomposition of the methyl orange was followed by measuring the absorption of its most intensive peak (464 nm).

## Electronic supplementary material

Supporting information
