# Supplementary material for: Photocatalytic hollow TiO2 and ZnO nanospheres prepared by atomic layer deposition
Source: Sci Rep. 2017 Jun 28;7:4337. doi: 10.1038/s41598-017-04090-0 (PMC5489517; doi:10.1038/s41598-017-04090-0)
Supplement: Supplementary file 1 — Supporting information [file 41598_2017_4090_MOESM1_ESM.pdf]

SUPPORTING INFORMATION OF THE MANUSCRIPT

Photocatalytic hollow TiO_2 and ZnO nanospheres prepared by atomic layer deposition

Nóra Justh[1], László Péter Bakos[1*], Klára Hernádi[2], Gabriella Kiss[2], Balázs Réti[2], Zoltán Erdélyi[3], Bence Parditka[3] and Imre Miklós Szilágyi[1,4]

[1]Department of Inorganic and Analytical Chemistry, Budapest University of Technology and Economics, H-1111 Budapest, Hungary

[2]Department of Applied and Environmental Chemistry, University of Szeged, H-6720 Szeged, Hungary

[3]Department of Solid State Physics, University of Debrecen, H-4026 Debrecen, Hungary

[4]MTA-BME Technical Analytical Chemistry Research Group, H-1111 Budapest, Hungary

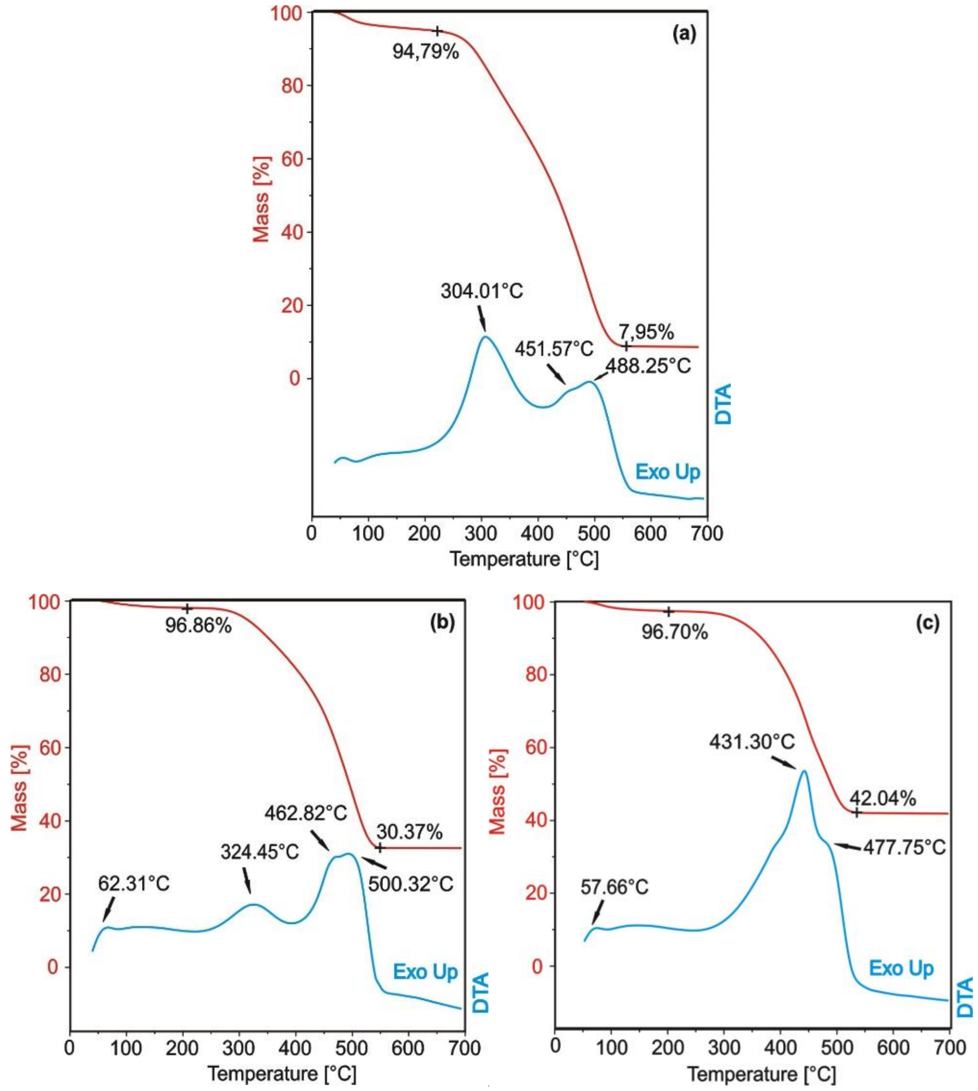

Figure S1. TG/DTA data of the carbon core removal in air. (**a**) is C-TIO_2-80C, (b) is C-TIO_2-250C and (**c**) is C-ZnO-250C.

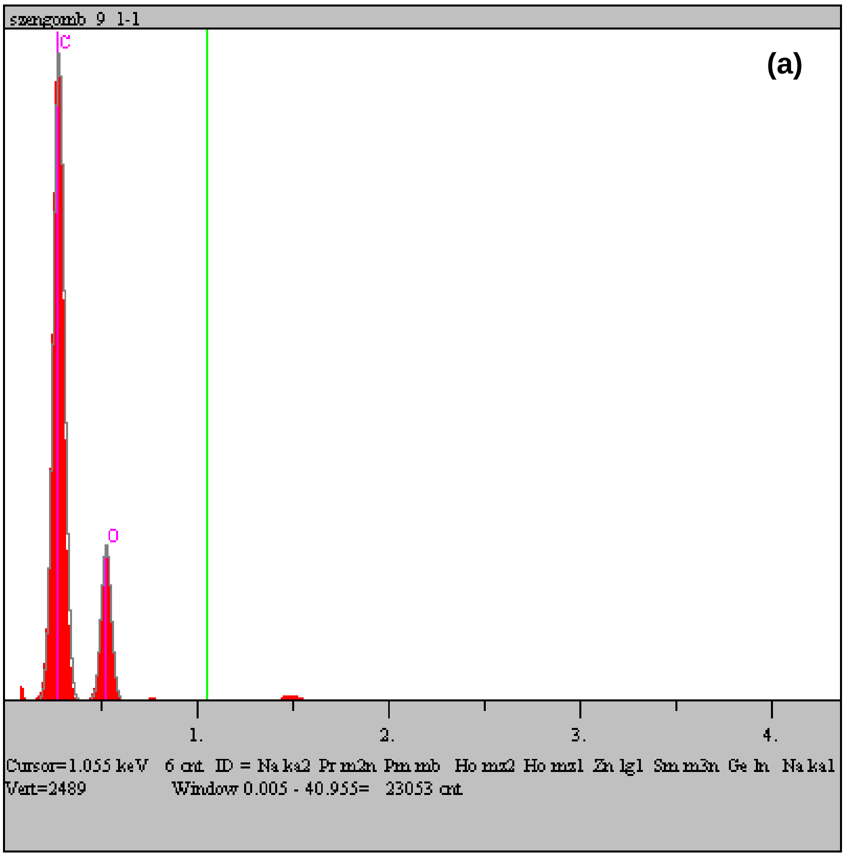

szengomb 9 1-1
(a)
C
O
1.
2.
3.
4.
Cursor=1.055 keV 6 cnt ID = Na ka2 Pr m2n Pm mb Ho mz2 Ho mz1 Zn lg1 Sm m3n Ge ln Na ka1
Vert=2489 Window 0.005 - 40.955= 23053 cnt

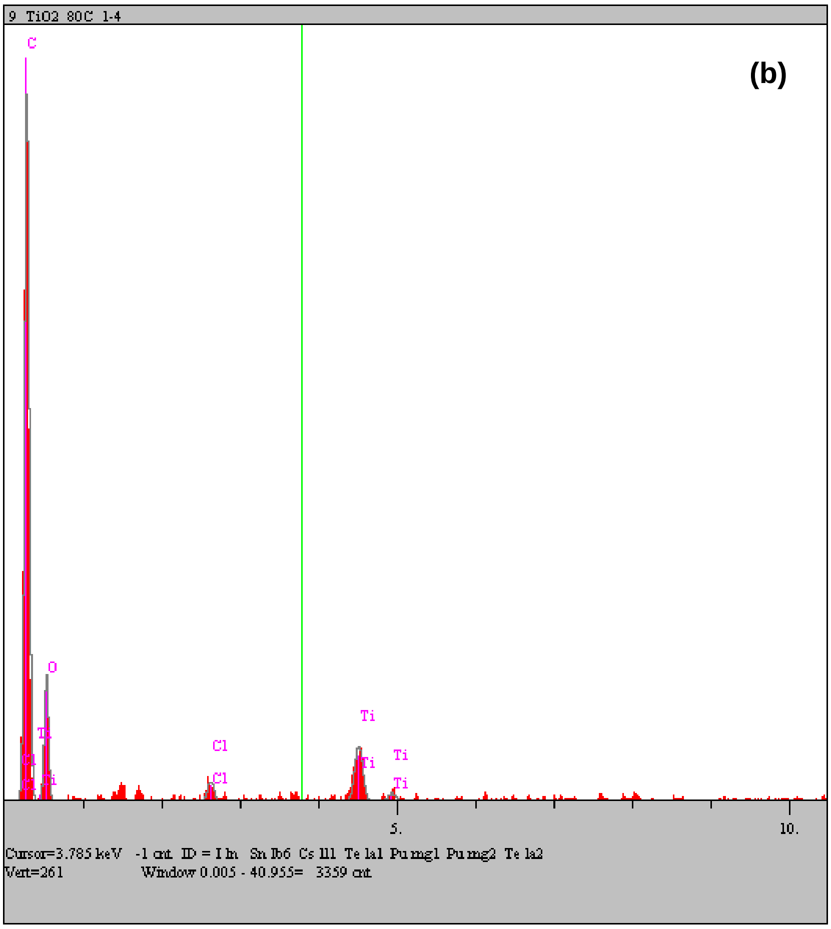

9 TiO2 80C 1-4
(b)
C
O
Ti
Cl
Ti
5.
10.
Cursor=3.785 keV -1 cnt ID = I ln Sn lb6 Cs ll1 Te la1 Pu mg1 Pu mg2 Te la2
Vert=261 Window 0.005 - 40.955= 3359 cnt

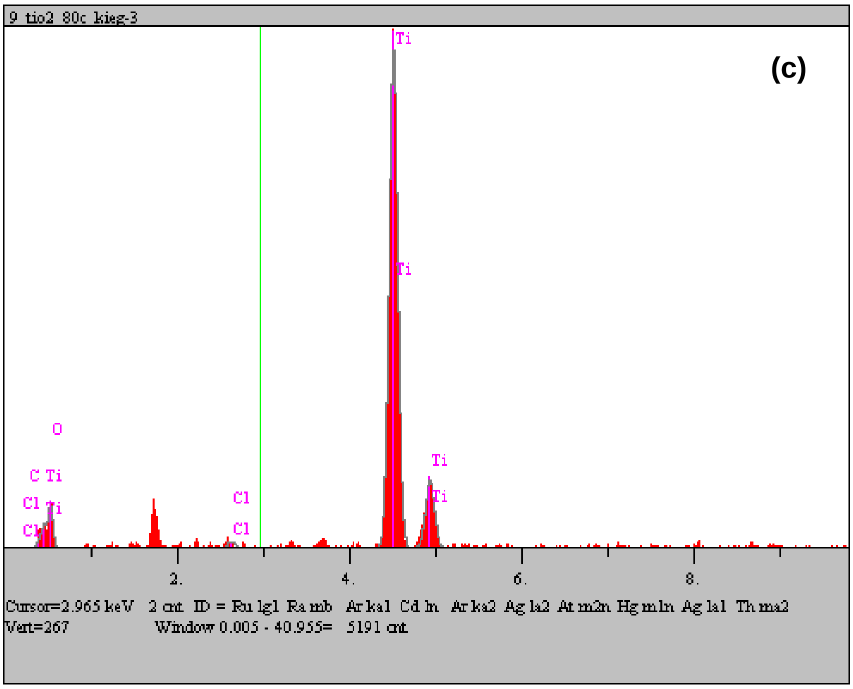
9 tio2 80c kieg-3
(c)
Ti
Ti
O
C Ti
Cl
Ti
Cl
Cl
Cl
Ti
Ti
2.
4.
6.
8.
Cursor=2.965 keV 2 cnt ID = Ru lg1 Ra mb Ar ka1 Cd ln Ar ka2 Ag la2 At m2n Hg m1n Ag la1 Th ma2
Vert=267 Window 0.005 - 40.955= 5191 cnt

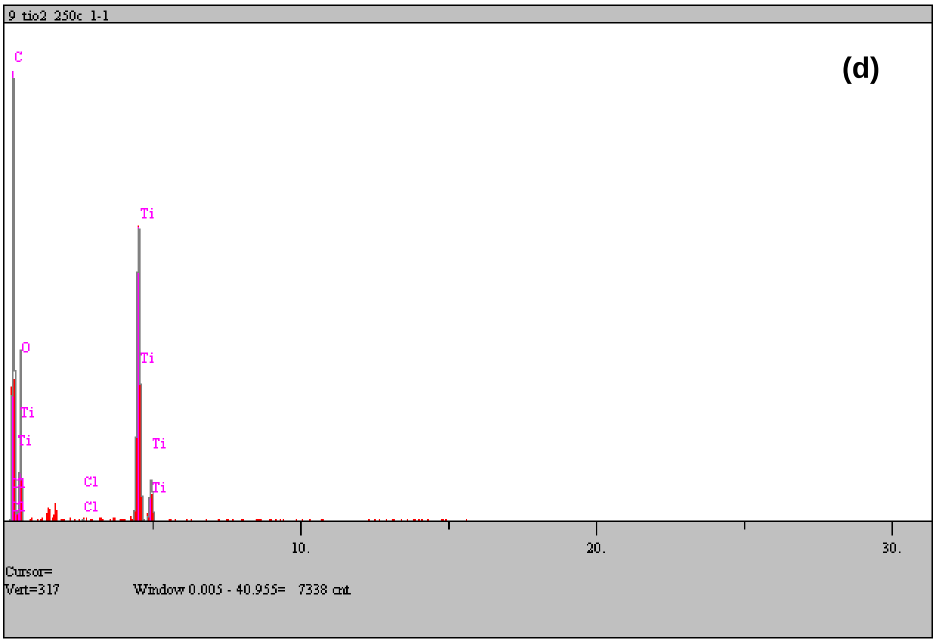
9 tio2 250c 1-1
(d)
C
Ti
O
Ti
Ti
Ti
Cl
Cl
Ti
Ti
10.
20.
30.
Cursor=
Vert=317 Window 0.005 - 40.955= 7338 cnt

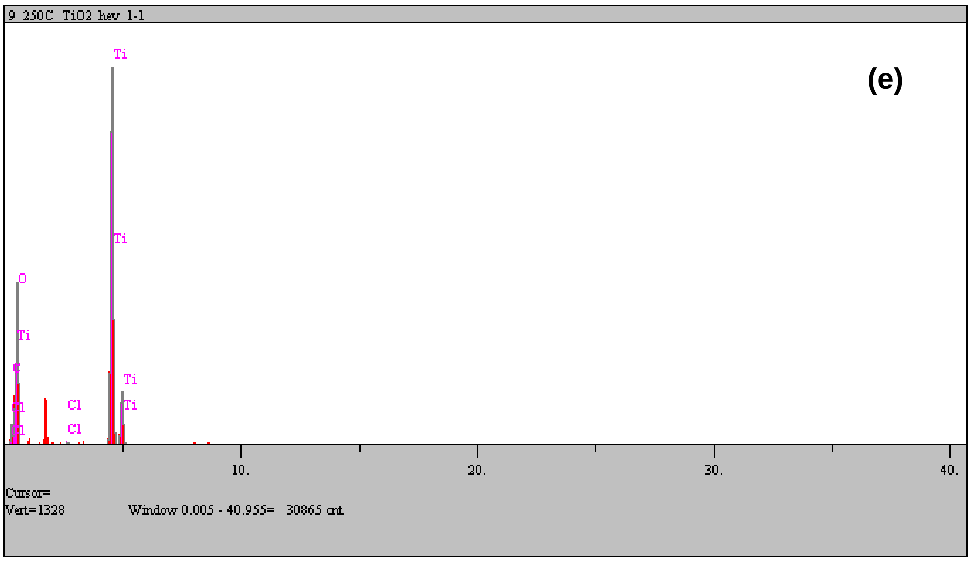
9 250C TiO2 hev 1-1
(e)
Ti
Ti
O
Ti
Ti
Ti
Cl
Cl
10.
20.
30.
40.
Cursor=
Vert=1328 Window 0.005 - 40.955= 30865 cnt

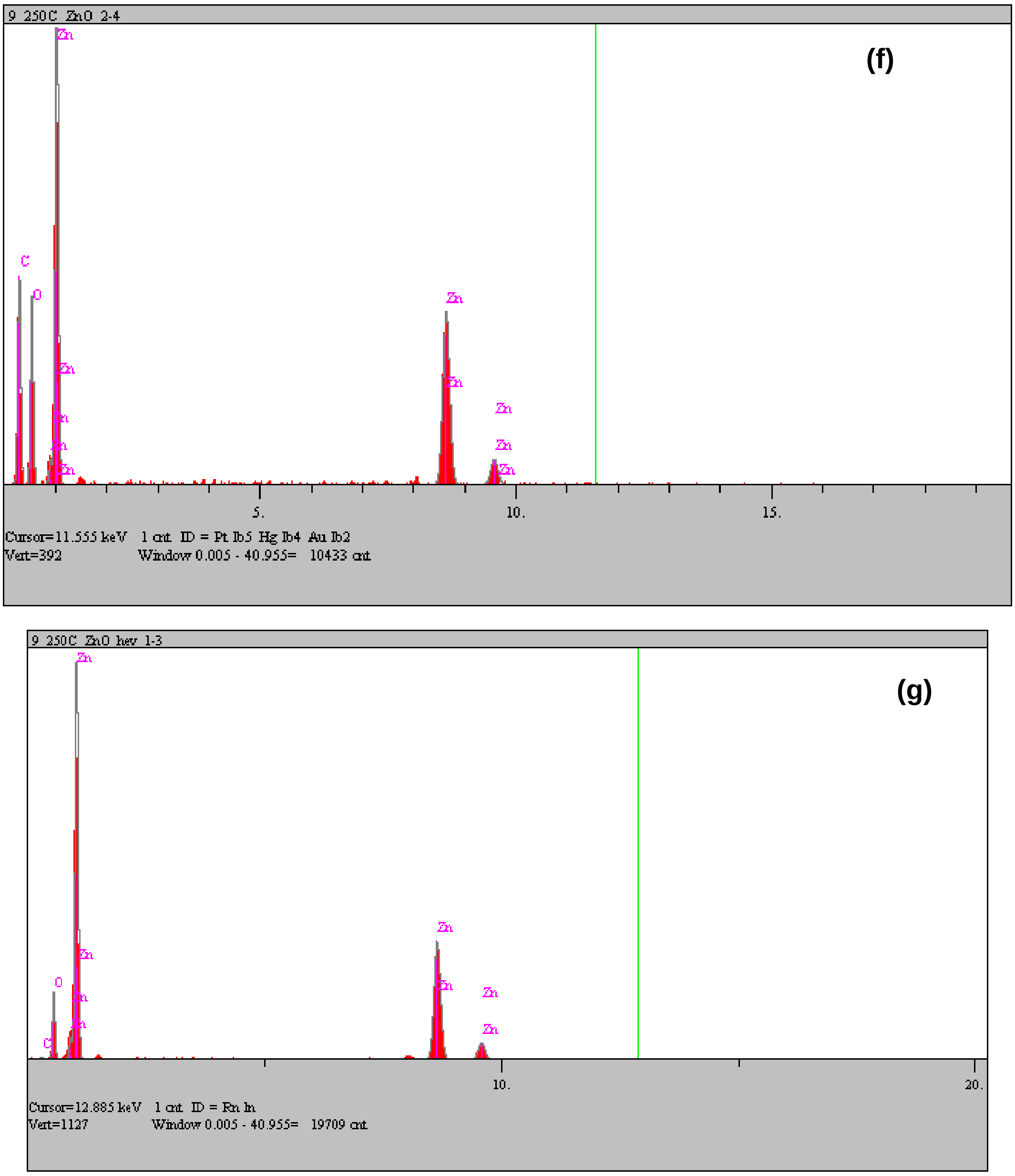

Figure S2. Example EDX spectra for each specimen. (**a**) pure carbon spheres, (**b**) C-TiO_2-80C, (**c**) H-TiO_2-80C, (**d**) C-TiO_2-250C, (**e**) H-TiO_2-250C, (**f**) C-ZnO-250C and (**g**) H-ZnO-250C

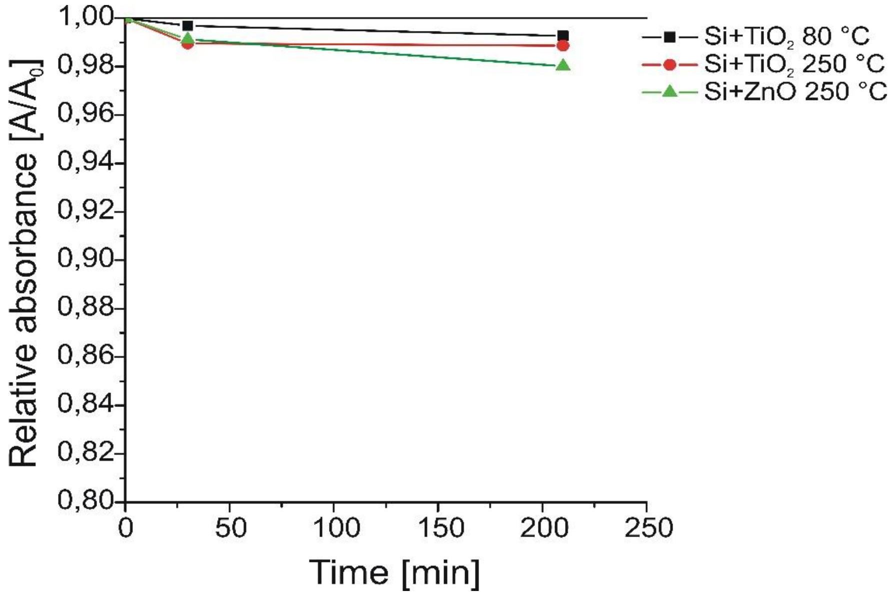

Figure S3. Analysis of the photocatalytic activity of the oxides deposited on silicon wafers.

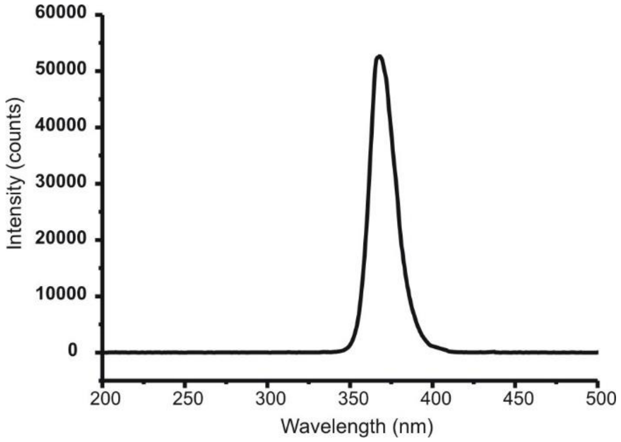

Figure S4. Spectrum of the Osram UV lamps.

Sample	Thickness
Si+TiO_2 at 80 °C	34.0 nm
Si+TiO_2 at 250 °C	38.6 nm
Si+ZnO at 250 °C	62.4 nm

Table S1. Thicknesses of the oxides deposited on flat silicon wafers measured by UV-VIS reflection
